# Microbiome diversity in mosquitoes and sand flies: implications for vector competence

**DOI:** 10.1186/s13071-025-06964-z

**Published:** 2025-09-24

**Authors:** Gnanasekar Ragini, Mahima K. Mani, Rohit Sharma, Nikhil Bharadwaj, Muthukumaravel Subramanian, Shriram Ananganallur Nagarajan, Manju Rahi

**Affiliations:** 1 Division of Vector Biology and Control, ICMR-Vector Control Research Centre, Medical Complex, Indira Nagar, Puducherry, 605006 India; 2 Pondicherry University, R. Venkataraman Nagar, Kalapet, Puducherry, 605 014 India; 3 Academy of Scientific and Innovative Research (AcSIR), Ghaziabad, 201002 India

**Keywords:** Gut microbiome, Vector competence, Mosquito, Sandflies, Diversity, Microbiome

## Abstract

Mosquitoes and sand flies are the most important vectors of several human diseases. A comprehensive analysis of the diversity and composition of the microbiome in mosquitoes and sandflies is important. It emphasises shared traits and distinctive differences between these vector species. Recent findings have demonstrated that physiological, environmental and ecological factors influence the diversity of these microbial communities. A deeper understanding of the functional roles of specific microbial taxa, such as their ability to modulate host immune responses or directly interact with pathogens, reveals exciting opportunities for innovative vector management strategies. These strategies could leverage microbiome manipulation to disrupt the transmission of disease-causing agents. However, despite notable advancements, critical gaps remain in unravelling the precise mechanisms by which these microbiome compositions influence vector competence. Ultimately, this understanding can be leveraged to harness the potential of microbiome-based interventions in reducing the burden of vector-borne diseases. This review explores the intricate relationships between microbial communities and key vectors, highlighting how these interactions influence the dynamics of pathogen transmission.

**Graphical abstract:**

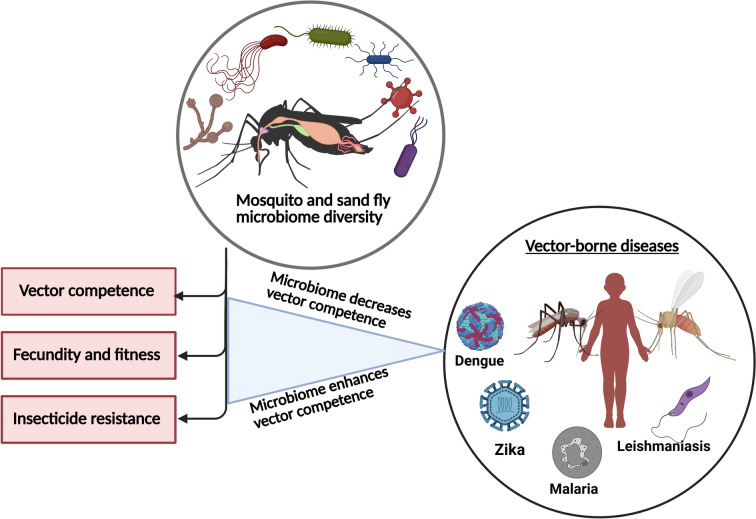

## Background

More than 700,000 people die from vector-borne diseases (VBDs) each year, which account for more than 17% of all infectious diseases worldwide [1]. Vectors such as mosquitoes, sand flies and ticks transmit infectious pathogens (viruses, parasites and bacteria) between humans or from animals to humans, causing vector-borne diseases. Many of these vectors are blood-sucking insects that ingest pathogens from an infected host during a blood meal and later transmit them to new hosts. These diseases are more prevalent in tropical and subtropical areas, where they disproportionately affect economically disadvantaged populations [2].

The most important arthropod vectors are mosquitoes, which transmit various diseases that affect millions of people worldwide. *Anopheles*, *Aedes* and *Culex* are the three main genera of vector mosquitoes that transmit different vector-borne diseases. About 249 million people worldwide are affected by malaria-causing parasites that are transmitted by *Anopheles* mosquitoes [3]. *Aedes* mosquitoes primarily transmit arboviruses, such as dengue virus (DENV), Rift Valley fever virus (RVF), Zika virus (ZIKV), chikungunya virus (CHIKV) and yellow fever virus (YFV), predominantly affecting urban and peri-urban populations [4, 5]. *Culex* mosquitoes transmit pathogens, including West Nile virus (WNV) and Japanese encephalitis virus (JEV), are involved in the transmission of lymphatic filariasis, a disease that leads to lifelong morbidity and disability [6]. Another important group of insect vectors is the phlebotomine sandflies, which transmit diseases such as leishmaniasis, bartonellosis, sandfly fever, summer meningitis, vesicular stomatitis and Chandipura virus encephalitis [7–10]. The increasing prevalence of VBDs can be attributed to factors such as insufficient healthcare infrastructure in endemic regions, parasite resistance to drug therapies, vector resistance to insecticides and failures in vector surveillance and control [11–16].

**Fig. 1:**
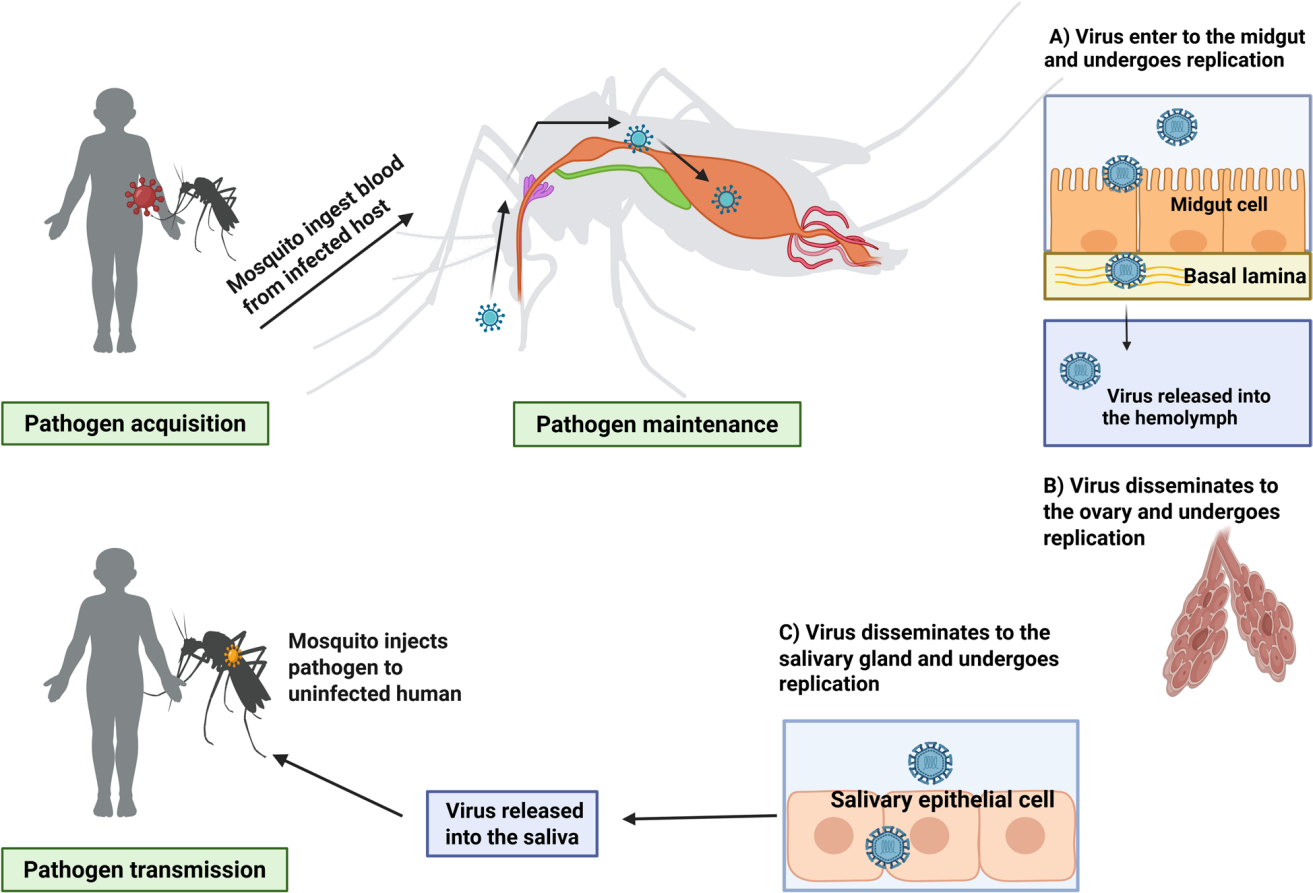
The replication, maintenance and transmission of infectious agents by the mosquito vector. Figure created with BioRender.com

The microbiome is a community of microorganisms, including bacteria, viruses, protozoans and fungi, residing within and playing a significant role in shaping host biology, influencing processes such as nutrition, reproduction, metabolism, immunity and vector competence [17]. Vector competence refers to a vector’s ability to acquire, maintain and transmit a pathogen, such as a virus, bacterium or parasite, to hosts [18] (Fig. 1). By influencing pathogen survival and modulating immune responses, the microbiome plays a crucial role in determining and developing vector competence. It can either enhance or reduce a vector’s ability to transmit diseases [19]. This variation in vector competence arises through mechanisms such as immune response activation, competition for resources or the production of antiviral compounds [20]. For example, microorganisms can prime the immune system, enhancing the expression of immune-related genes and the production of antimicrobial peptides [21]. The microbiota also aids in nutrient absorption and energy production, thereby improving metabolic efficiency [22]. It also plays a role by competing with pathogens for resources and limiting their ability to establish infections [23]. Additionally, the microbiome can produce secondary metabolites responsible for inhibiting pathogen replication [21, 24]. Notably, *Enterobacter ludwigii*, *Pseudomonas rhodesiae* and *Vagococcus salmoninarum* isolated from *Aedes albopictus* have been shown to produce antiviral compounds capable of inhibiting La Crosse virus replication in vitro [25]. Furthermore, certain microbiota-derived molecules, such as prodigiosin, a red tripyrrole pigment synthesised by several bacterial species, including *Serratia marcescens*, have demonstrated inhibitory effects against *Trypanosoma cruzi* and *Plasmodium* species under in vitro conditions [26].

The microbiome composition and acquisition in vector organisms are shaped by a combination of biotic and abiotic influences, including the genetic traits of both the host and the microbes, as well as environmental conditions [27–29]. Consequently, microbiome profiles can vary significantly between individuals, developmental stages, species and geographical regions [30, 31]. Climatic conditions directly influence the availability of microbes in the breeding habitats of insect vectors. A change in climatic conditions, such as temperature and humidity, may influence microbial diversity in the environment. Therefore, vector populations growing in different geographical areas may acquire variable compositions of microbiomes [30, 32, 33]. For example, a study conducted in China revealed significant variation in gut microbiota across eight regions in various mosquito species, including *Ae. albopictus*, *Aedes galloisi*, *Culex pallidothorax*, *Culex pipiens*, *Culex gelidus* and *Armigeres subalbatus* [34]. Similar regional differences were observed in the microbiomes of *Culex tritaeniorhynchus* and *Culex orientalis* in the Republic of Korea [35]. In another study, global variations in the microbiomes of *Ae. albopictus* and *Aedes japonicus* were reported in eight countries, including Europe, the USA and Japan [36]. Likewise, the composition of sand fly microbiomes was associated with different geographical regions in Iran and Tunisia [37, 38]. These variations likely contribute to phenotypic differences between the vector hosts [39].

Microbes located in critical tissues, such as the midgut or salivary glands, may directly interact with pathogens, influencing their survival, replication or transmission. Meanwhile, microbes in other tissues may exert indirect effects on the vector’s ability to transmit diseases (Tables 1, 2). It has been demonstrated that the insect midgut microbiome can influence susceptibility to dengue virus serotype 2 (DENV-2) by inhibiting prohibitin on the midgut surface of mosquitoes [40]. Bacterial species such as *Asaia* spp., *S. marcescens*, *Chromobacterium* sp. *Panama* (Csp_P) and *Enterobacter* spp. in the mosquito midgut are associated with enhancing the immunity of vectors [41–43]. A reduced microbiome enhances *Plasmodium* oocysts in various anopheline mosquitoes [21, 44]. In sand flies, midgut bacteria have also been found to provide essential nutrients that facilitate *Leishmania* attachment to the gut wall [45]. Furthermore, microbes in the salivary gland can either inhibit or promote the colonisation of pathogens within these glands [46, 47].

**Table 1:** Microbiome diversity in different tissues of mosquitoes

Tissue (mosquitoes)	Microbiomes	Reference
Midgut	*Enhydrobacter*, *Aeromonas*, *Serratia*, *Ralstonia*, *Lactobacillus*, *Pseudomonas*, *Streptococcus*, *Rubrobacter*, *Anaerococcus*, *Methylobacterium*, *Turicibacter*, *Elizabethkingia*, *Corynebacterium*, *Stenotrophomonas*, *Rhizobium*, *Sphingobacterium*, *Wolbachia*, *Chryseobacterium*, *Pectobacterium*, *Asaia*, *Gluconobacter*, *Staphylococcus*, *Citrobacter*, *Klebsiella*, *Enterobacter*, *Escherichia*, *Proteus*, *Acinetobacter*, *Saccharomyces*, *Enterococcus*, *Kytococcus*, *Lactococcus*, *Kocuria*, *Alloiococcus*, *Sphingomonas*, *Acidovorax*, *Aquabacterium*, *Bosea*, *Flectobacillus*, *Hydrogenophaga*, *Isosphaera*, *Roseomonas*, *Sphingopyxis*, *Zoogloea*, *Delftia*	[46, 50, 51, 57–65]
Salivary gland	*Enhydrobacter*, *Aeromonas*, *Serratia*, *Ralstonia*, *Lactobacillus*, *Pseudomonas*, *Streptococcus*, *Rubrobacter*, *Anaerococcus*, *Methylobacterium*, *Turicibacter*, *Elizabethkingia, Nitrospira*, *Corynebacterium*, *Acinetobacter*, *Staphylococcus*, *Bacillus*, *Massilia*, *Delftia*, *Shigella*, *Cutibacterium*, *Atopococcus*, *Stenotrophomonas*, *Rhizobium*, *Sphingobacterium*, *Wolbachia*, *Chryseobacterium*, *Pectobacterium*, *Aeromonas*, *Enterobacter*, *Paracoccus*, *Enterococcus*, *Pantoea*, *Lysinibacillus*, *Brevibacterium*, *Klebsiella*, *Saccharomyces*, *Kocuria*, *Lactococcus*, *Escherichia*, *Burkholderia*, *Asaia*, *Sphingomonas*, *Cupriavidus*	[46, 50, 51, 57, 58, 66]
Malpighian tubules	*Pseudomonas* *Wolbachia*	[67] [20]
Reproductive organs	*Ochrobactrum*, *Pseudomonas*, *Sphingomonas*, *Stenotrophomonas*, *Acinetobacter*, *Elizabethkingia*, *Asaia*, *Wolbachia*, *Staphylococcus*, *Corynebacterium*, *Geobacillus*, *Micrococcus*, *Kocuria*, *Streptococcus*, *Stenotrophomonas*, *Aerococcus*, *Serratia*, *Klebsiella*, *Pantoea*, *Microbacterium*, *Phytobacter*, *Spiroplasma*	[50, 52, 53, 55, 68, 69]

**Table 2:** Microbiome diversity in different tissues of sand flies

Tissue sand fly	Microbiomes	Reference
Gut	*Enterobacter*, *Aeromonas*, *Erwinia*, *Pseudomonas*, *Acinetobacter*, *Propionibacterium*, *Trabulsiella*, *Wolbachia*, *Asaia*, *Serratia*, *Stenotrophomonas*, *Enhydrobacter*, *Chryseobacterium*, *Edwardsiella*, *Microbacterium*, *Staphylococcus*, *Escherichia*, *Proteus*, *Klebsiella*, *Kluyvera*, *Leminorella*, *Pantoea*, *Providencia*, *Rahnella*, *Shigella*, *Tatumella*, *Yersinia*, *Kocuria*, *Streptococcus*, *Methylobacterium*, *Bacillus*, *Ochrobactrum*, *Cutibacterium*, *Spiroplasma*, *Propionibacterium*, *Diplorickettsia*, *Rickettsia*, *Rickettsiella*	[37, 185, 190, 196, 198–204]
Salivary gland	*Enterobacter*, *Spiroplasma*, *Ralstonia*, *Acinetobacter*, *Reyranella*, *Undibacterium*, *Bryobacter*, *Corynebacterium*, *Cutibacterium*, *Psychrobacter*, *Wolbachia*	[200]
Ovary	*Bradyrhizobium*, *Pseudomonas*, *Ochrobactrum*	[197]

Currently, controlling vectors is the main strategy for managing vector-borne diseases (VBDs). Understanding the complex relationships between insect vectors and their associated microorganisms is an important first step toward developing innovative approaches to reduce pathogen transmission and improve VBD control. This review focuses on how the microbiome affects vector biology and competence. It also explores new applications of microbiome research in creating effective strategies to combat VBDs.

### Mosquito and microbiome: a complex interaction

The microbiome of mosquitoes exhibits significant variation across different tissues, including the midgut [48, 49], salivary glands [50–52], reproductive tracts [53–55] and cuticle surfaces [56]. This tissue-specific variation or tissue tropism reflects the distinct microenvironments and functional roles of each anatomical site within the mosquito host.

### The gut microbiome

**Fig. 2:**
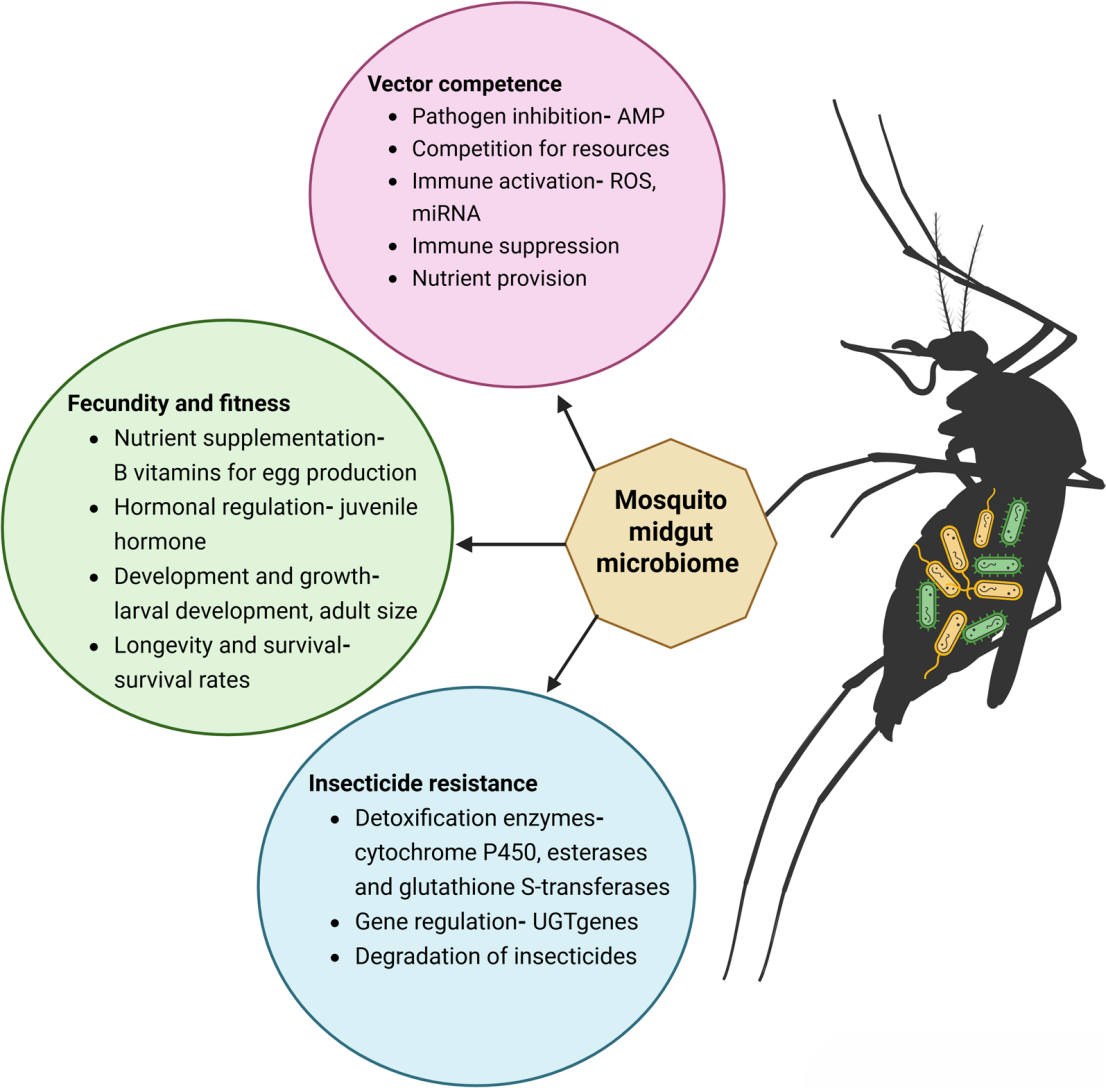
The various roles of midgut microbiota in mosquito biology. Figure created with BioRender.com

The gut microbiota, also known as the gut microbiome, includes bacteria, archaea, fungi, viruses and parasites that reside in the digestive tracts of animals. These microorganisms play a crucial role in various functions, such as digesting food, producing vitamins, regulating the immune system, nutrient absorption, regulating water balance, expelling waste and behavioural modulation [70–73]. The roles of microbiomes in insect physiological processes have been studied. For example, microbes such as *Elizabethkingia anophelis*, *Enterobacter* spp. and *Serratia* spp. are involved in blood meal digestion [74, 75]. Other microbes, such as *Wolbachia* spp., *S. marcescens*, *Chromobacterium* sp. and *Asaia* spp. are involved in the immune system regulation [21, 76–79]. *Escherichia coli* in the mid-gut microbiome is known to be involved in riboflavin (vitamin B2) synthesis, facilitating energy metabolism, oxidative stress response, blood digestion and egg production in mosquitoes (Fig. 2) [80]. The presence of microbial communities within the digestive tracts of mosquitoes has been recognised for many years [81]. According to research conducted in the early 1900s, mosquitoes’ digestive tracts contain a community of extracellular microorganisms that form the gut microbiota in both larval and adult stages [82]. However, detailed investigations into the composition and functionality of these microbiota have emerged mainly over the past decade [83]. The diverse microbiome of mosquitoes may vary between species and developmental stages. Various breeding sites may have different compositions of microbes, which are acquired during the larval stage. However, adult mosquitoes can acquire different microbial communities through nectar, blood meals or by imbibing water from their breeding sites when they emerge [84–87]. For example, variations in microbiomes in different developmental stages have been seen in *Anopheles gambiae*, *Ae. albopictus* and *Cx. pipiens* [83, 84, 88].

### The role of mosquito gut microbiota

In mosquitoes, functional investigations have mostly examined how gut microbiota affects vector competence. Additionally, research has provided valuable insights into nutrient acquisition, development, insecticide resistance and oviposition behaviour.

### Mosquito fitness, oviposition and hatchability

The microbiome is essential for mosquito development and significantly influences host immune signalling pathways and lifespan. For example, the presence of bacteria such as *E. coli* in mosquito larvae appears to play a critical role in larval development into adulthood. These microorganisms may induce hypoxia within the larval gut by scavenging oxygen in the gut lumen, creating anoxic conditions. Research suggests that these anoxic conditions serve as developmental cues, facilitating the transition of larvae to the pupal stage [89]. Early research on mosquito larvae showed that increased mortality and delayed growth resulted from a decrease in microbial diversity of microorganisms in the aquatic environment [81, 90]. A study by Coon et al. [84] utilized surface sterilization techniques on mosquito eggs to generate axenic larvae of *Ae. aegypti*, *Aedes atropalpus* and *An. gambiae*; they were verified to be free of gut microbiota through polymerase chain reaction (PCR) and culture-based methods. Interestingly, when given a diet that was sufficiently nutritious and germ-free, larvae of every species failed to moult and died at the first instar stage. However, axenic larvae’s growth into adulthood was fully restored when they were colonised by different individual members of the gut community or given normal gut microbiota [84]. Riboflavin, a vitamin B2, is produced naturally by microbiota in the midguts of mosquitoes. It is important for the development of larvae and adults. Recent research has shown that riboflavin mediates this association since axenic larvae can mature into adults when reared in environments that maintain this vitamin and have an anoxic midgut [91] (Fig. 2). These studies collectively highlight gut microbiota’s critical role in mosquito development.

The presence of bacteria also appears to influence egg hatching. Studies have shown that *Aedes aegypti* eggs with higher concentrations of bacteria (e.g., *Enterobacter cloacae* and *Bacillus* species) on their surfaces hatch more rapidly than those with lower bacterial loads [92]. Furthermore, experiments comparing bacterial-rich and germ-free environments reveal significant differences in egg-hatching rates [93]. This phenomenon may be attributed to bacterial production of signalling molecules such as carboxylic acids and methyl ester metabolites, which not only promote egg hatching but also act as oviposition attractants for *Ae.* *aegypti* [94]. Additionally, bacterial metabolic byproducts in the aquatic environment may create conditions that are conducive to egg hatching, thereby further enhancing reproductive success. According to the study, ovipositing females preferred rearing water from larvae infected with *Ascogregarina taiwanensis* or *Candida* near *pseudoglaebosa* rather than distilled water or rearing water from uninfected larvae [95].

### Acquisition and digestion of food

Mosquitoes primarily obtain nutrients from plant nectar and blood meals. While males feed exclusively on nectar, females consume tryptophan in the nectar for energy and blood to support egg development [96, 97]. As holometabolous insects, mosquitoes undergo complete metamorphosis, with distinct larval and adult life stages separated by pupation [98]. During their aquatic larval stages, mosquitoes generally feed as detritivores before pupating. Adult gut microbiota consists of bacteria that were transferred from the larval stage and obtained through mating, sugar-eating and water consumption [99, 100]. The gut microbiota plays a vital role in larval development, with living bacteria activating signalling pathways involved in nutrient sensing. The gut microbiota in *Ae. aegypti* larvae promote development by providing essential nutrients such as riboflavin and inducing hypoxia-related signalling pathways, crucial for moulting and growth [80]. A study by Coon et al.[89] identified that bacterial respiration reduces gut oxygen levels, triggering hypoxia-inducible pathways that drive moulting and growth in mosquito larvae. The gut microbial diversity and composition can be influenced by the vector’s host-feeding preferences, which may affect the vector’s ability to acquire and transmit pathogens [101]. Antibiotic treatments using carbenicillin and tetracycline have been shown to significantly reduce the number of culturable gut bacteria in adult female *Ae. aegypti*. This reduction results in a statistically significant decrease in the ability to lay eggs and slight delays in the digestion of blood meals [74]. Gut microbes are also important in autogenous mosquitoes for nutrient acquisition, which can produce eggs without blood-feeding [85]. For instance, *Enterobacteriaceae*, a prominent bacterial family in *Ae. albopictus* metabolises fructose, a key sugar in nectar, to generate nutrients beneficial to the host [102].

These findings highlight the crucial role of gut microbiota in both digestion and reproductive processes in mosquitoes, suggesting potential avenues for further research into mosquito physiology and vector control strategies.

### Insecticide resistance

The term ‘insecticide resistance’ refers to an insect’s ability to tolerate exposure to insecticides. Several biological mechanisms can lead to insecticide resistance, including metabolic, target-site and behavioural resistance [103]. Metabolic resistance involves the increased detoxification of the insecticide through increased production of metabolic enzymes in insects such as esterases, cytochrome P450 monooxygenases and glutathione-*S*-transferases (GSTs) [104]. Previous research on insecticide resistance in mosquitoes has mostly concentrated on their genes. The connection between mosquitoes’ symbiotic bacteria and their insecticide resistance has garnered more attention in recent years. Gut microbes play a significant role in influencing insecticide resistance in mosquitoes by modulating the detoxification and metabolism of chemical compounds [105, 106]. These microorganisms can also produce enzymes such as esterases (GSTs) and cytochrome P450s, which are known to degrade or modify insecticides, thereby reducing their toxicity (Fig. 2) [107]

**Fig. 3:**
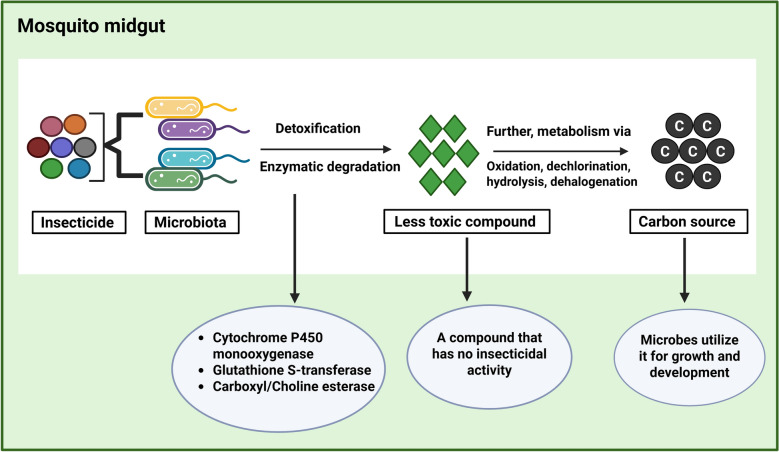
This image illustrates how the microbiome detoxifies the insecticide and further metabolises it

In *Cx. pipiens pallens* strains resistant to deltamethrin, *Wolbachia* was found to be more prevalent, indicating its role in enhancing resistance to this insecticide [108]. Likewise, *Klebsiella pneumoniae* enrichment was found by metagenomic sequencing of *Anopheles albimanus* strains resistant to fenitrothion [56, 109, 110]. Other microbes, including *Bacillus cereus* [111], *Acidomonas* sp. [112], *Staphylococcus aureus* [113], *Pseudomonas* *fluorescens*, *E. cloacae* [114] and *Aspergillus niger* [115] also exhibit high potential for breaking down deltamethrin. Additional research suggests that the use of fenitrothion significantly boosts *Pseudomonas* and *Flavobacterium* abundance [116, 117]. By breaking down fenitrothion into 3-methyl-4-nitrophenol, a substance with no insecticidal properties, these bacteria may further convert it into a carbon source and utilise it for development [118] (Fig. 3). According to Zhang et al., *Pseudomonas* is involved in the metabolism of pyrethroids and organophosphates [119]. The symbiotic bacteria can promote resistance to organophosphate insecticides by increasing the metabolic activity of *An. stephensi* in response to chemicals [120]. Interestingly, several bacterial species in the mosquito gut can directly metabolise insecticides, converting them into less harmful compounds, which are then further metabolised and excreted. These findings reveal a distinct correlation between midgut microbiota and insecticide resistance in mosquitoes, with potential implications for their vector competence.

### Impact of microbiome in modulating vector competence

The term ‘vector competence’ describes a vector’s innate ability to acquire, maintain and transmit a pathogen, comprising several barriers and complex processes within the vector [121]. When mosquitoes feed on blood, they can ingest pathogens from host vertebrates. Components of the mosquito immune system are crucial for defence against arboviruses and *Plasmodium* parasites. The peritrophic matrix and midgut epithelium are two significant barriers that prevent the entry of different pathogenic microbes [122]. The microbial community associated with mosquito larvae has a significant effect on the adult mosquitoes immunological responses and vector competence. This can either enhance or inhibit their ability to transmit diseases.

### Microbiota and arboviruses

Microbiota-arbovirus interactions appear to be a key factor in establishing arboviral infection and transmission [25, 123]. For example, when the *An. gambiae* microbiota was eliminated through antibiotic treatment, and the mosquitoes were less susceptible to O’nyong’nyong virus (ONNV) viral infection [124]. Conversely, *Ae. aegypti* became more susceptible to DENV infection when exposed to the mosquito-associated fungus *Talaromyces* sp. which suppresses the expression of digestive enzymes and trypsin activity in the mosquito gut [125]. Additionally, differences in microbiome composition have been noted between DENV-susceptible and DENV-resistant *Ae. aegypti* populations, including variations in bacterial genera such as *Acinetobacter* and *Pseudomonas*, with certain taxa potentially influencing susceptibility [126, 127].

In *Culex* mosquitoes, infection with WNV has been linked to an increase in bacterial diversity [128]. Similarly, CHIKV infection in *Ae. albopictus* reduces *Wolbachia* abundance and increases *Enterobacteriaceae* abundance. Instead, ZIKV infection changes the microbiome composition in *Ae. aegypti* [129, 130].

**Fig. 4:**
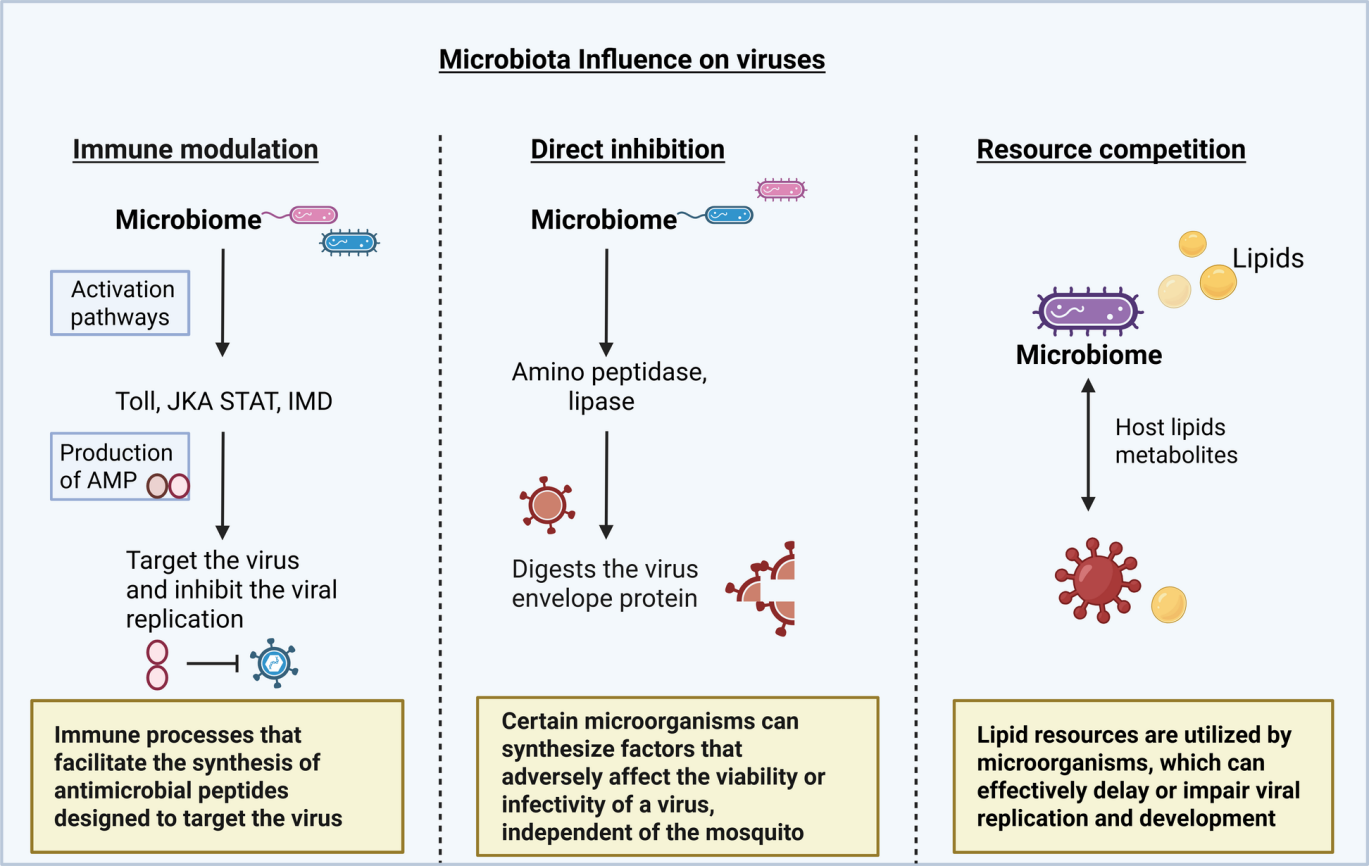
The three primary ways of the microbiota influencing arboviruses. Figure created with BioRender.com

The study on *Aedes* mosquitoes demonstrated that higher titres of dengue virus serotype 2 (DENV-2) were seen after treating *Ae. aegypti* with antibiotics. It has been demonstrated that anti-microbial peptide (AMP) and reactive oxygen species (ROS) expression are decreased when gut bacteria are absent (Fig. 2) [78, 131]. For instance, *Aedes* mosquito larvae exposed to *E. coli* at sub-lethal levels produce important immune components such as nitric oxide and AMP, which improve defence against subsequent infections [132]. Furthermore, it has been demonstrated that infection with certain strains of *Wolbachia* can disrupt DENV and ZIKV infections in *Ae. aegypti* [133]. *Enterobacter hormaechei* B17 secretes sphingosine, a metabolite that blocks the virus and efficiently reduces ZIKV infection in cell cultures and *Ae. aegypti* [42]. Csp_P is a notable bacterium that has been shown to decrease DENV infection in *Ae. aegypti* by generating an amino-peptidase that breaks down the DENV envelope protein (Fig. 4) [41, 78]. Moreover, Csp_P produces the anti-parasitic protein rhomidepsin, which inhibits *Plasmodium falciparum* infection in *An. gambiae* [122].

However, several gut bacteria make mosquitoes more susceptible to arboviruses. For instance, *Serratia odorifera*, which is present in mosquitoes’ guts, makes *Ae. aegypti* more susceptible to DENV or CHIKV infection [40, 134]. This effect is mediated by the bacterial polypeptide P40, which interacts with the protein prohibitin, a factor related to arboviral infection in mosquito cells [40, 135]. Similarly, by generating a protein known as SmEnhancin, which breaks down the mucin layer of the mosquito midgut epithelium and increases susceptibility to infection, a strain of *S. marcescens* increases DENV infection in *Ae. aegypti* [136, 137]. This protein plays a critical role in the interaction between the mosquito vector and the host it bites. It aids in modulating the host’s immune response, creating an environment conducive to pathogen survival and transmission [137]. By suppressing certain immune pathways in the host, SmEnhancin facilitates the establishment and replication of DENV within the host after a mosquito bite [137].

Mosquitoes with higher levels of SmEnhancin expression may exhibit an enhanced ability to transmit pathogens, as they are more effective at suppressing host defences. Conversely, variations in SmEnhancin production, whether due to genetic differences or environmental factors, could influence the mosquito’s efficiency as a vector [137].

Further research is needed to elucidate the exact molecular structure and pathways involved in SmEnhancin activity. Understanding how environmental and ecological factors influence SmEnhancin expression in mosquito populations could also provide insights into the varying transmission dynamics of mosquito-borne diseases across regions. These findings highlight the complex relationships between mosquito microbiota and arboviruses, revealing both protective and enhancing roles of microbial species in viral infection and transmission.

### Insect-specific viruses (ISVs)

Insect-specific viruses (ISVs) are non-pathogenic to vertebrates due to their inability to replicate in mammalian cells; however, these viruses can suppress the replication of arboviruses in mosquitoes/sandflies [138–140]. These viruses share many similarities with established arboviruses and can be transmitted vertically [141]. They are classified within major viral families such as Flaviviridae, Bunyaviridae, Negeviruses, Togaviridae, Reoviridae, Mesoniviridae and Rhabdoviridae [142, 143]. Experimental studies have shown that the replication of DENV and ZIKV can be suppressed in both *Ae. aegypti* and C6/36 mosquito cell lines when co-infected with an ISV known as the cell-fusing agent virus (CFAV) [144]. Likewise, the Nhumirim virus (NHUV) can reduce the replication of WNV, JEV and Saint Louis encephalitis virus (SLEV) in insect cell lines [145]. Furthermore, when mosquitoes were experimentally co-infected with NHUV and WNV, the dissemination rate of WNV was significantly reduced [146]. Prior infection with Eilat virus (EILV) reduces the virus titres of CHIKV, western equine encephalitis virus (WEEV), Venezuelan equine encephalitis virus (VEEV) and eastern equine encephalitis virus (EEEV) in C7/10 cells [142, 147]. The difference in the relative abundance of ISV families in field-caught *Ae. aegypti* was between the DENV-infected and non-infected mosquitoes. Additionally, Phasi charoen-like phasivirus (PCLV) was observed frequently in DENV-infected mosquitoes [148].

### Microbiota and plasmodium

Protozoa of the genus *Plasmodium* cause malaria. In total, four species are known to cause disease mainly in humans: *P. falciparum*, *Plasmodium vivax*, *Plasmodium ovale* and *Plasmodium malariae*. Other *Plasmodium* species, such as *Plasmodium knowlesi* and *Plasmodium simium*, primarily infect reptiles, birds and other mammals but can also infect humans. For example, human cases of *P. knowlesi* malaria have been reported in Malaysia, Thailand, the Philippines, Myanmar and several other Southeast Asian countries [149]. Additionally, a human case of *P. simium* infection has been reported in Brazil [150]. The *Plasmodium* species is greatly influenced by the presence of a microbiome in the midgut. Studies have shown that reducing midgut bacterial populations through antibiotic treatment leads to an increased burden of oocysts and a higher prevalence of various human and rodent malaria parasites across different *Anopheles* species, the primary vectors of malaria [44, 151–154].

Recent research indicates that bacteria play a vital role in triggering a ‘priming’ immune response to *Plasmodium* in *An. gambiae*. For instance, during parasite invasion, bacteria-driven activation of a haemocyte differentiation factor promotes the transformation of haemocytes into granulocytes, as observed by Rodrigues et al. [155]. This increase in granulocytic haemocytes strengthens the mosquito’s defence against subsequent infections by *Plasmodium* or other pathogenic agents. The reintroduction of Gram-negative bacteria into mosquitoes has been found to reduce the intensity of *Plasmodium* infections in a dose-dependent manner [152, 156]. Bacteria commonly colonising *Anopheles* mosquitoes, such as *Comamonas* sp., *Acinetobacter*, *Pseudomonas*, *Pantoea*, *Serratia*, *Enterobacter* and *Elizabethkingia*, exhibit mosquito-independent inhibition of *Plasmodium* [21, 43, 78]. Furthermore, it has been demonstrated that infection with certain strains of *Wolbachia* can disrupt *Plasmodium* infections in *An. stephensi* [157]. Antibiotic elimination of the microbiota led to increased levels of tryptophan and its metabolites, including 3-hydroxykynurenine (3-HK), which damaged the peritrophic matrix and promoted *Plasmodium berghei* infection [158].

Several bacterial species also produce protective factors that act against various stages of the parasite’s lifecycle. Using bacteria-free culture supernatants, such as Csp_P and *Serratia*, has shown the ability to block *Plasmodium* asexual and sporogonic stages. These inhibitory effects are likely mediated by antimicrobial compounds or enzymes secreted by the bacteria, which directly interfere with parasite development.

Therefore, midgut bacteria play a critical role in modulating mosquito vector competence for malaria parasites. They may achieve this through immune signalling pathways or direct interactions with the parasites, thereby limiting the mosquito’s ability to transmit malaria.

### Salivary gland microbiome

Salivary glands have an equally important function in the replication and transmission of pathogens as the midgut [159]. The salivary viral titre is an ideal indicator of vector competence [160]. However, sufficient viral replication within the salivary glands is essential before the virus can be transmitted through saliva to a new host [161]. Even salivary glands are an important dissemination site where arboviruses can replicate; they must cross the salivary gland infection and escape barriers for successful release into the saliva for further transmission [162]. Salivary gland microbiomes in insects can significantly influence their behaviour by interacting with the host’s physiology and neural pathways. Insects often rely on their microbiomes for essential functions such as digestion, immunity and even reproduction; however, emerging research shows that microorganisms in the salivary glands also affect behaviour. These microbiomes can influence insect feeding preferences, social behaviours and even their ability to transmit pathogens [22, 163]. Understanding these relationships is crucial, as it not only provides insight into insect physiology but also offers potential strategies for insect vector control or disease prevention.

### The diversity of the microbiome in the salivary gland

The diverse range of microbiota that make up a mosquito’s salivary gland microbiome is crucial for both the mosquito’s function and its ability to transmit disease [51]. This microbiome can influence vector competence by interacting with pathogens, affecting their survival, replication and transmission [164]. The composition of the salivary gland microbiome varies among mosquito species, but commonly identified genera include *Acinetobacter*, *Pseudomonas* and *Serratia* [165]. For instance, *Acinetobacter* and *Pseudomonas* are predominant in the salivary glands of *Ae. aegypti* and *Ae. albopictus*, which are key vectors for diseases such as dengue, Zika and chikungunya [46]. In *Anopheles* mosquitoes, bacteria such as *S. marcescens* have been shown to modulate the mosquito’s ability to transmit *Plasmodium* parasites [47]. Moreover, field studies on *Anopheles darlingi* indicate significant differences in the salivary gland microbiota between lab-reared and wild populations, with genera such as *Wolbachia* and *Asaia* playing a role in modulating mosquito–pathogen interactions [57].

The salivary gland microbiome is also implicated in physiological processes such as saliva production, which is vital for blood feeding and pathogen transmission. Mosquito saliva facilitates the replication and dissemination of viruses within vertebrate hosts. Recent research highlights that the saliva of anopheline mosquitoes harbours bacteria that are introduced into mammalian hosts during blood feeding. Surprisingly, a study has shown that *P. berghei* and certain bacteria can successfully colonize the tissues of mammalian hosts through mosquito saliva [47]. This dual colonization highlights the complexity of host, pathogen and microbiome interactions, which can potentially influence disease outcomes and shape pathogen evolution within vertebrate hosts.

### Ovary microbiome

The ovaries are critical sites for virus replication, facilitating the vertical transmission of pathogens from the mother to oocytes [166]. Similarly, bacteria present in the ovaries can be transmitted vertically from one generation to the next [167]. The ovary microbiome in vector mosquitoes plays a vital role in their reproductive success, vector competence and overall physiology. The extracellular bacterium *Asaia* can vertically spread after colonising the *Anopheles* mosquito ovaries [168, 169]. *Wolbachia*, an intracellular bacterium, not only infects somatic tissues but also invades the germ cells in the ovaries reliably, enabling vertical transmission [167, 170, 171]. *Serratia* AS1, another extracellular bacterium, also persistently colonises *Anopheles* ovaries and is passed from females to their offspring. Notably, *Serratia* AS1 is also found in the accessory glands of male *Anopheles* mosquitoes, enabling its transmission through mating [68]. Although research on microbial sexual transmission in mosquitoes is limited, bacteria such as *Asaia* and *Serratia* have been identified in the reproductive organs of both *Anopheles* and other mosquito species, suggesting their potential role in venereal transmission during copulation [68, 172].

Research indicates that mosquito species such as *Ae. aegypti* and *Ae. albopictus* harbour distinct bacterial communities in their ovaries, which differ significantly from their gut microbiota [53]. Studies show that the ovary microbiota includes genera such as *Serratia*, *Klebsiella*, *Wolbachia* and *Asaia*, with species-specific variations. For instance, *Wolbachia* is prominent in *Ae. albopictus* but less so in *Ae. aegypti*, where *Serratia* and *Enterobacter* are more common [173–175]. These microbes are acquired from the environment and then selectively partitioned among different tissues, including ovaries, where they may influence mosquito fertility and the transmission of pathogens such as DENV and ZIKV [176].

Understanding the bacterial composition of mosquito ovaries is crucial for elucidating the role of these microbes in host–pathogen interactions. This knowledge can reveal whether ovary-associated bacteria influence susceptibility or vector capacity through mechanisms such as immune modulation, the production of bioactive molecules or the establishment of reproductive isolation, particularly by endosymbionts such as *Wolbachia* [177, 178].

### Sand fly and microbiome

Sand flies are tiny insects that belong to the family Psychodidae [8]. They act as vectors for several diseases, most notably leishmaniasis, which is caused by protozoa of the genus *Leishmania* [179]. Sand flies thrive in warm, humid environments and are mainly active during the evening and night-time [180]. Once the parasites enter the host, they invade and multiply within host cells, leading to different clinical manifestations, resulting in mucocutaneous, visceral and cutaneous leishmaniasis [181].

**Fig. 5:**
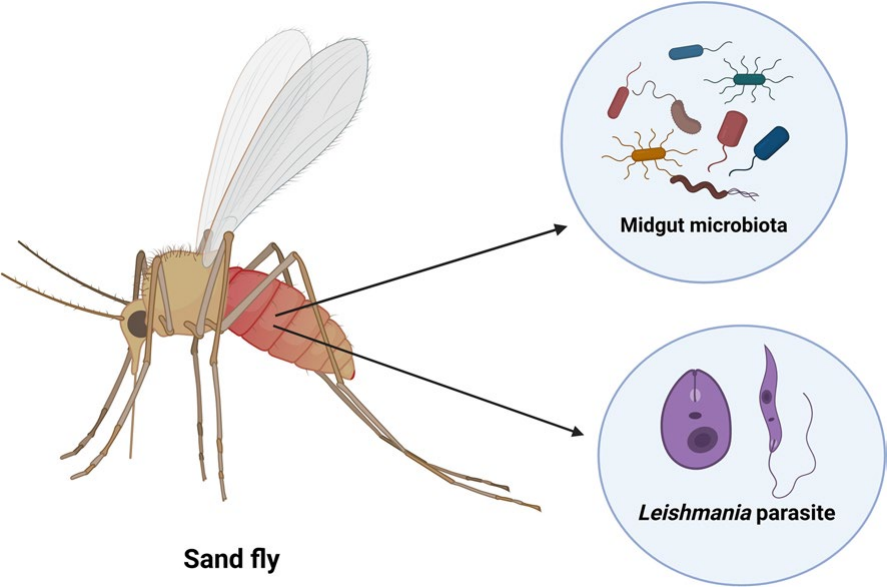
Overview of sand fly microbial community and pathogen association. Figure created with BioRender.com

In addition to leishmaniasis, sandflies can transmit other pathogens, including viruses (phleboviruses) and bacteria (*Bartonella*), causing sandfly fever and bartonellosis, respectively. The microbiomes of sand flies, particularly those in their gut, play a crucial role in influencing their vector competence [182]. Gut microbial communities can modulate the development of parasites such as *Leishmania*, either enhancing or inhibiting their growth, thereby affecting the sand fly’s ability to transmit pathogens [183, 184] (Fig. 5).

### The composition and diversity of the microbiome in sand flies

Similar to in other Diptera, the microbiota of the sand fly is a dynamic community that primarily originates from its environment. Its composition changes as it progresses through various developmental stages, profoundly influenced by the environments and food sources it encounters [185–187]. Sand fly eggs are typically laid in environments such as soil, animal burrows and decaying wood [188, 189], which are known for their rich microbial diversity. This highlights the significant relationship between the microbial communities present in the guts of sand flies and their natural breeding habitats [190].

Few studies have reported that genera such as *Enterobacter* and *Acinetobacter* are present in both the adult and larval stages of *Pintomyia evansi*, indicating that flies may acquire microbes from their surroundings through feeding or that certain bacteria can persist transstadially, being passed from larvae to adults [191, 192]. For obvious reasons, wild-caught sand flies exhibit greater gut bacterial diversity compared with their lab-reared counterparts, likely due to the restricted diet in laboratory settings [193, 194]. Based on a meta-analysis, the majority of bacteria found in Old World sand fly species are members of the phyla *Proteobacteria* and *Firmicutes* [192]. Additionally, bacteria of the *Actinobacteria* phylum were also reported. In *Lutzomyia* sp., bacteria belonging to the phylum *Proteobacteria* were abundant, followed by *Firmicutes* and *Actinobacteria* [192]. *Methylobacterium*, a dominant inhabitant of plant phyllospheres, is consistently present in the midgut of *Pi. evansi*, suggesting a plant-based diet. The presence of *Methylobacterium* in *Pi. evansi* was more prominent in engorged females, indicating potential interactions between plant-derived microbes, blood nutrients and the parasites transmitted during blood feeding [195]. Hassan et al. previously identified a diverse microbiome community within the midgut of *Phlebotomus papatasi*. This community includes bacteria such as *Shigella sonnei*, *Alcaligenes faecalis*, *Serratia liquefaciens*, *Haemophilus parainfluenzae*, *Bacillus thuringiensis* and *Listeria seeligeri* [186]. The bacterium *Ochrobactrum* sp. was found in the midguts of *P. duboscqi*, a known vector of *L. major* in sub-Saharan Africa [185]. The composition and diversity of gut bacteria in sand flies are influenced by their feeding habits and infection status. Sucrose-fed *Lu. longipalpis* has the most diverse microbiome [196], but this diversity decreases with blood meals, most likely owing to the antimicrobial effects of blood. However, microbial diversity recovers after blood digestion. This pattern is also seen in *Lu. intermedia*, with gravid females showing similar microbial diversity to non-fed females [197]. Across all groups (sucrose-fed, blood-fed and infected), the genera *Stenotrophomonas*, *Serratia*, *Pantoea* and *Erwinia* are consistently present [197].

### Role of the gut microbiome in sand flies

The gut microbiome plays a vital role in various physiological and ecological processes in sand flies, contributing to functions such as development, pathogen resistance, nutrient acquisition and oviposition [7, 187]. Research has shown that bacteria play a crucial role in the oviposition behaviour of sand flies. The oviposition preferences of *Lu. longipalpis* were assessed using both sterile and non-sterile rabbit faeces. The findings revealed that gravid females laid the majority of their eggs on the non-sterile substrate, demonstrating a strong attraction to rabbit faeces containing a live microbial community. This suggests that the presence of bacteria significantly influences the oviposition site selection of the sand fly [187]. Gut bacteria are essential for the proper development of sand fly larvae. In the study by Peterkova-Koci et al., larvae fed on sterile rabbit faeces showed delayed progression to adulthood and reduced survival compared with those given sterilised faeces supplemented with *Rhizobium radiobacter* or *Bacillus* species. Interestingly, *R. radiobacter* was more effective in facilitating larval development than *Bacillus* spp. suggesting that certain bacteria play a key role in providing essential nutrients, such as amino acids and vitamins [187].

Notably, *L. mexicana*-infected sand flies demonstrated that survival rates were high when challenged with *S. marcescens* compared with uninfected flies [205]. The persistence of neutrophils at bite sites, where they shield captured parasites and exacerbate disease, is a notable characteristic of *Leishmania* transmitted by vectors. Studies have shown that, alongside the *Leishmania* parasites, gut microbes from the sand fly are also introduced into the host’s skin. These microbes activate the inflammasome, rapidly releasing interleukin (IL)-1β, which sustains neutrophil infiltration. This suggests a shared mechanism of the microbiota that contributes to the severity of the infection [182]. These results suggest a potential mutualistic relationship, where *Leishmania* may protect the sand fly from bacterial infection or modulate the immune response, benefiting both the host and the parasite.

**Fig. 6:**
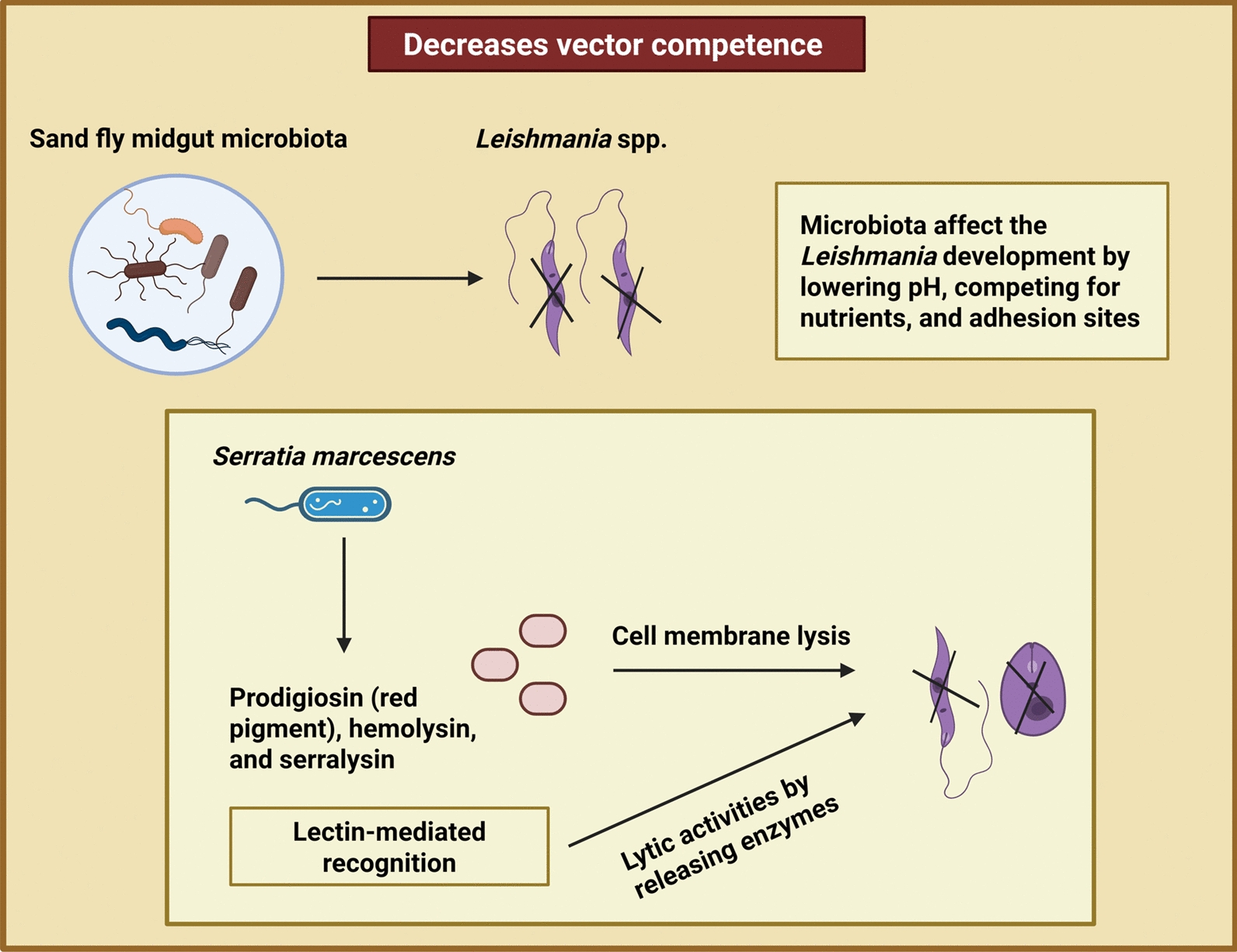
The role of sand fly midgut microbiota, particularly *S. marcescens*, in modulating the *Leishmania* parasite development. Figure created with BioRender.com

In female sand flies, *Leishmania* parasites and various bacterial species co-exist in the gut. Several studies hypothesise that the sand fly midgut bacteria can affect parasite development by lowering the intestinal pH and competing with the parasite for nutrients and adhesion sites in the vector’s gut (Fig. 6) [198]. Studies have shown that certain gut microbiota components can interfere with *Leishmania* development. For example, *S. marcescens*, has been reported to negatively affect* L. infantum* by producing prodigiosin, a compound that induces lysis of the parasite’s cell membrane (Fig. 6) [206, 207]. Supporting this, an in vivo study demonstrated that *Lu. longipalpis* fed on a microbial suspension containing *Asaia* sp., *Pseudozyma* sp. or *Ochrobactrum intermedium* exhibited reduced infection rates of *L. mexicana* in sand flies [205].

Interestingly, research by Hassan et al. showed that *P. papatasi* treated with antibiotics were more susceptible to *L. major* infection compared with untreated controls, indicating that gut symbionts contribute to resistance against *Leishmania* [186]. However, in contrast to these findings, emerging evidence suggests that the native gut microbiota may be crucial for *Leishmania* survival and development [196]. Disruption of this microbiota through antibiotic treatment has been shown to impair the parasite’s growth and hinder its differentiation into the infectious metacyclic form. This highlights the complex and potentially supportive role that the microbial community plays in the life cycle of *Leishmania* within its sand fly vector.

**Fig. 7:**
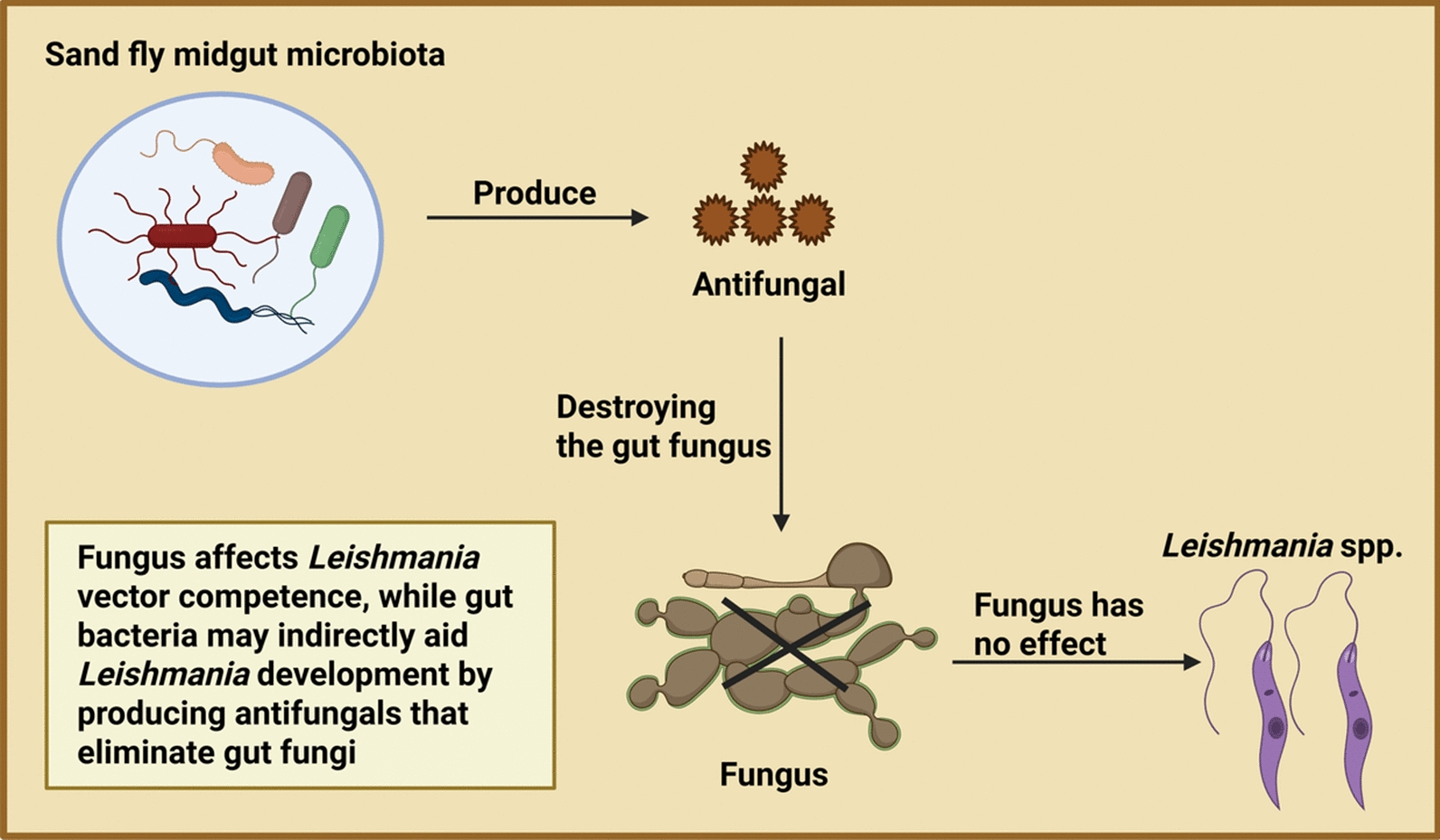
The role of antifungal compounds produced by sand fly midgut microbiota in enhancing *Leishmania* development. Figure created with BioRender.com

Gut bacteria might indirectly support *Leishmania* development by producing anti-fungal compounds that eliminate fungi from the gut. This is significant, as sand flies infected with fungi are less competent vectors than those with fungi-free guts [208] (Fig. 7).

## Conclusions

The microbiome of mosquitoes and sand flies plays a crucial role in shaping their vector competence, thereby influencing the transmission dynamics of numerous vector-borne pathogens. These complex microbial communities, influenced by host genetics, environmental factors and pathogen exposure, interact intricately with their insect hosts and can influence their fitness, development and fecundity. The microbiome also contributes to insecticide resistance and helps regulate the immune response, thereby influencing disease transmission. Certain microbial taxa are known to interact directly with pathogens, facilitating conditions that enhance their survival and transmission. In other cases, the microbiome modulates host immune pathways, potentially promoting vector longevity. Targeting these complex microbe, host and pathogen interactions presents a promising and sustainable strategy for future vector control. Innovations in microbiome engineering, such as enhancing beneficial microbial communities to reduce vector competence or introducing genetically modified microbes to inhibit pathogen transmission, may offer eco-friendly alternatives to conventional chemical control methods. Future research should focus on unravelling the molecular mechanisms by which microbiomes influence vector competence. Longitudinal studies tracking microbiome composition across developmental stages, species and environmental contexts could provide valuable insights into the dynamic interplay between vectors and their microbial communities. Integrating microbiome research with cutting-edge fields such as genomics, immunology and ecological modelling will be crucial for translating these discoveries into effective vector control tools. Collaborative, interdisciplinary efforts will be essential to fully realise the potential of microbiome-based strategies for reducing the global burden of VBDs. A deeper understanding of microbial communities and their roles in the fitness and competence of the vector can be beneficial for controlling VBDs.

## Data Availability

Data supporting the main conclusions of this study are included in the manuscript.

## Abbreviations

DENV: Dengue virus
RVF: Rift Valley virus
ZIKV: Zika virus
CHIKV: Chikungunya virus
YFV: Yellow fever virus
WNV: West Nile virus
JEV: Japanese encephalitis virus
VBDs: Vector-borne diseases
ONNV: O’nyong’nyong virus
PCR: Polymerase chain reaction
ROS: Reactive oxygen species
AMP: Anti-microbial peptide
Csp_P: *Chromobacterium* Sp.* Panama*
GSTs: Glutathione-*S*-transferases
CFAV: Cell-fusing agent virus
NHUV: Nhumirim virus
